# Supra-Renal Melasma

**Published:** 1869-07

**Authors:** J. C. Peters


### Supra-Renal Melasma.

BY J. C. PETERS, M. D.

Case 3 was that of a convict who had been in Sing Sing for eleven years. In attempting to escape he was shot by a keeper above the anterior superior spinous process of the ilium, and died on the eighth day. The peritoneum was filled with blood and pus, but the ball could not be found. The skin was *bronzed* all over as in Addison's disease, but no supra-renal capsules could be hunted up; on the other hand granular kidneys were found as in Bright's disease.

As this patient was strong enough to attempt to escape from prison, he could hardly have been suffering from the excessive debility and extreme anæmia which attends well developed Addison's disease. As the supra-renal capsules could not be found they were doubtless completely atrophied. Although it is supposed that they perform some important office in the animal economy, and minister in some way to the elaboration of the blood, in common, possibly, with the other ductless glands, viz.: spleen, thymus and thryroid bodies, still all that we know is, that they seem more important to the young subject than to the adult. For, they are always larger than the kidneys in the embryo, about an equal size in very young children, and only about the twentieth as large in the adult. Indeed, as is well known, they are sometimes so small in the latter that they can hardly be found. When they are healthy and of the natural size, they have a yellowish red color, and are from one and one-half to two inches in length, and rather more than an inch in breadth, but are so thin and flat as only to weigh one to two drachms. Their great peculiarity is, that their nerves are disproportionately large and numerous, and are chiefly derived from the solar, though somewhat from the renal plexuses. In a fatal case of Addison's disease Drs. Aitken and Monro found that the sympathetic nerves from the lesser *splanchnic* were greatly increased in size, as were the ganglia of the *solar plexus* towards the side of the supra-renal capsule most diseased or in contact with it. The substance of these nerves presented a light rosy hue as if under the influence of vascular excitement. Aitkin says there are good grounds for believing that Addison himself entertained the belief that death in these cases was due eventually to the implication of the ganglionic nerves.

However this may be, gastro-intestinal disturbance prevails during life, and a condition of the mucous membranes is found after death, which Aitken supposes may be associated in pathology with the disease of the capsules and of the nerves. The stomach is often ecchymosed and Brunner's glands in the duodenum and the solitary glands in the lower part of the ilium and colon are apt to be very prominent. The mucous membrane of the mouth may be thin, pale and bloodless, although the labial and buccal glands are enlarged. The stomach and substance of the intestinal tube may be uniformly thin throughout. The solitary gastric glands may be remarkably prominent, while the mucous membrane is wasted and atropic. The villi of the ilium and jejunum may be remarkably attenuated; the tubular glands almost entirely gone and their place supplied by granular amorphous material.

In some cases the heart undergoes fatty degeneration; the spleen becomes much enlarged, the kidneys become pale and finally reach the last stage of fatty degeneration; the mesenteric glands become enlarged, and tubercle or cancer may be found in some other organ.

It will now be very easy to understand the symptomatology of the disease, viz.: a morbid state, establishing itself with extreme insidiousness, whose characteristic features are general languor and extreme prostration, expressed by loss of muscular power, weakness of pulse, remarkable feebleness of the heart's action, breathlessness and faintness from slight exertion, dimness of sight, functional weakness and irritability of the stomach, etc. But it is not so easy to account for the peculiar discoloration or *bronzing* of the whole surface of the body; although, as Aitken says, it is true that in malarious, malignant and cachectic diseases, it is not unusual, but rather the rule, for the serum of the blood to assume a dark and dirty hue, so that ultimately the skin closely approaches in color that which obtains in jaundice, differing, sometimes, only from it in being more lurid and dusky. It is believed that the hue of the skin, which becomes of so dark a tinge in some organic diseases, is due to the admixture of morbid matters absorbed from the seat of local mischief, and which so tinges the serum of the blood that the *rete mucosum* is rendered dark. Thus in cancer there is a dirty straw yellow color of the skin, while the cancer juice appears as a viscid, whitish and creamy or yellowish fluid, which may be squeezed or scraped in considerable abundance from the cut surface of the cancerous part. *Chloasma* or *maculæ hepaticæ* are frequently caused by uterine irritation or torpor, induced by impending catamenia, or amenorrhœa, before the system is relieved, or the blood purified by the natural flux (maculæ amennorrhoicæ;) or when suppression takes place as in pregnancy (maculæ gravidarum.) Chloasma may last through a considerable period of pregnancy, and only cease after the completion of parturition and the attendant lochial discharges. Other exciting causes of chloasma are gastro-intestinal, or hepatic irritation, or a morbidly sensitive state of the solar plexus of nerves. The color of syphilitic maculæ is peculiar; for it has been compared by some to that of dull metallic copper, by others to that of the lean part of ham. There is contamination of the blood, and altered serum is poured out in various parts of the skin. Even Ephelis or sunburn, and Lentigo or freckle, are caused by the pouring out of an excess of serum, with some hæmatin, into the rete mucosum of the skin from the irritation caused by great exposure to the rays of a burning sun. Rayer speaks of the discolored or mottled appearance seen upon the legs and thighs of women who sit over charcoal braziers, under the name of *ephelis ignealis, or umbrosa.*

Wilson says one of the functions of the skin is the formation of pigment, and one of the natural changes occurring at puberty, is the alteration of the skin of the sexual apparatus to a brown color, more or less dark in different individuals, which in rare instances may increase to a deep black. The alteration of color which takes place in the areola around the nipple in pregnant women is an analogous change, which in one very rare instance extended over the whole breast, and up to the throat, and down to the thighs. This disappeared in about a year. Such cases come under the head of *Nigritis,* or augmentation of skin-pigment; but all of them arise from some contamination of the blood, or irritation of the skin, which leads to the pouring out of hæmatin or some other coloring matter into the rete mucosum, causing a more or less transient or permanent discoloration of the surface.

The supra-renal capsules may suffer from many forms of disease; but, according to Dr. Wilks in the true morbus Addisonii the organs get enlarged and changed into a semi-translucent gray colored, soft and homogeneous material; which afterwards degenerates into a yellowish, white, opaque matter, and subsequently softens into a putty like substance, or dries up into a chalky mass. The other diseases of the capsules do not produce morbus Addisonii.

*Treatment.*—The management of the local disease is similar to that which obtains in the treatment of tubercle and scrofula. If the symptoms be referred to some failure of nervous force acting on the heart, induced by injury to the ganglionic system of nerves, then the most powerful tonics, such as citrate of iron and strychnine, extract of nux vomica, with quinine and iron, used freely and aided by beef-tea, milk punch, made with one-third lime water and two-thirds milk, with a sufficiency of spirits, either Jamaica rum, old brandy or good whisky, or egg nogg, etc., etc.

When the treatment of the disease is commenced sufficiently early, and the above tonics, stimulants and nutrients are given abundantly, it is very possible that the preparations of manganese may facilitate a cure which would otherwise be impracticable.—*Medical Gazette.*

